# Zolpidem is a potent stoichiometry-selective modulator of α1β3 GABA_A_ receptors: evidence of a novel benzodiazepine site in the α1-α1 interface

**DOI:** 10.1038/srep28674

**Published:** 2016-06-27

**Authors:** Ahmad Tarmizi Che Has, Nathan Absalom, Petra S. van Nieuwenhuijzen, Andrew N. Clarkson, Philip K. Ahring, Mary Chebib

**Affiliations:** 1 Faculty of Pharmacy, The University of Sydney, Sydney, New South Wales, 2006, Australia; 2 Department of Anatomy, Brain Health Research Centre and Brain Research New Zealand, School of Medical Sciences, University of Otago, Dunedin, 9054, New Zealand

## Abstract

Zolpidem is not a typical GABA_A_ receptor hypnotic. Unlike benzodiazepines, zolpidem modulates tonic GABA currents in the rat dorsal motor nucleus of the vagus, exhibits residual effects in mice lacking the benzodiazepine binding site, and improves speech, cognitive and motor function in human patients with severe brain injury. The receptor by which zolpidem mediates these effects is not known. In this study we evaluated binary α1β3 GABA_A_ receptors in either the 3α1:2β3 or 2α1:3β3 subunit stoichiometry, which differ by the existence of either an α1-α1 interface, or a β3-β3 interface, respectively. Both receptor stoichiometries are readily expressed in *Xenopus* oocytes, distinguished from each other by using GABA, zolpidem, diazepam and Zn^2+^. At the 3α1:2β3 receptor, clinically relevant concentrations of zolpidem enhanced GABA in a flumazenil-sensitive manner. The efficacy of diazepam was significantly lower compared to zolpidem. No modulation by either zolpidem or diazepam was detected at the 2α1:3β3 receptor, indicating that the binding site for zolpidem is at the α1-α1 interface, a site mimicking the classical α1-γ2 benzodiazepine site. Activating α1β3 (3α1:2β3) receptors may, in part, mediate the physiological effects of zolpidem observed under distinct physiological and clinical conditions, constituting a potentially attractive drug target.

γ-aminobutyric acid receptors of type A (GABA_A_) are members of the Cys-loop family of ligand-gated ion channels that mediate most of the inhibitory neurotransmission in the central nervous system (CNS). These receptors are pentameric assemblies of individual subunits including α1-6, β1-3, γ1-3, δ, ε, π and θ. The majority of receptors are composed of α, β, and γ or δ subunits^1^,^2^,^3^,^4^. Depending on the subunit composition, the receptors are located either at the synapse (α1/2/3βγ2 receptors) where they are exposed to brief (ms) bursts of high concentrations of GABA when the neuron fires (termed phasic inhibition) or at extrasynaptic regions (α1/4/6β, α5βγ2 or α4/6βδ receptors) where receptors typically experience long lasting (min to hours) exposure to relatively low but consistent concentrations of GABA (termed tonic inhibition)^2^,^3^,^4^. The most abundant receptors are α1βγ2 receptors, and these are activated and modulated by a variety of pharmacologically and clinically unrelated agents including benzodiazepines, barbiturates, anaesthetics and neurosteroids, all of which bind at distinct binding sites located within the receptor complex^1^,^2^,^3^,^4^.

Zolpidem is an imidazopyridine, a non-benzodiazepine, that binds with high-affinity to α1 containing GABA_A_ receptors (α1βγ2)^5^,^6^ and with lower affinity to α2 or α3 containing receptors (α2βγ2 and α3βγ2)^7^,^8^. In contrast, zolpidem is significantly more efficacious at α2 or α3 than α1 containing receptors^9^. The binding site for zolpidem at these receptors is located at the interface between the principle side (+) of the α and the complementary side (−) of γ2 subunit^4^,^10^,^11^. When zolpidem binds to this site, there is a structural change to the protein complex resulting in an increase in GABA potency^4^. The effects of zolpidem can be competitively blocked by the neutral modulator flumazenil^12^.

Like many benzodiazepines, zolpidem exhibits sedative and hypnotic effects via α1 containing receptors, but unlike benzodiazepines, zolpidem reverses cognitive and motor deficits in neuropathological states, improves motor function in patients with Parkinson’s disease, progressive supranuclear palsy and stroke^13^,^14^,^15^,^16^,^17^. Studies also show that there are residual effects with zolpidem in the γ2^F77I^ mouse where binding by zolpidem to α(+)-γ2(−) is attenuated^18^,^19^. Further, zolpidem can modulate tonic GABA currents in the rat dorsal motor nucleus of the vagus^20^ and primary motor cortex^15^, implicating extrasynaptic receptors containing α5 and δ subunits. However α5 and δ containing receptors do not significantly respond to zolpidem^7^,^8^ and neither do γ1/3 containing receptors^21^,^22^. Thus we sought to determine whether binary αβ receptors are potential targets that mediate zolpidem’s atypical effects.

Binary αβ receptors lack the benzodiazepine α-γ2 interface and co-exist with classical GABA_A_Rs^23^,^24^,^25^. The physiological role of these receptors remains elusive^25^,^26^,^27^, due to a lack of pharmacological tools that differentiate these receptors from their ternary counterparts. Interestingly, there are reports that certain benzodiazepines are able to enhance GABA actions at these receptors by binding to sites other than the classical α-γ2 interface^28^,^29^,^30^.

In pentameric αβ receptors, the third subunit is replaced with either an α1 or a β3 subunit leading to two distinct receptors that differ in subunit stoichiometry, 2α:3β^31^,^32^ or 3α:2β^33^. The consequence of this is that 3α:2β receptors contain an α-α interface whereas 2α:3β receptors contain a β-β interface, that are clearly distinct. Drugs that selectively bind to either the α-α or β-β interface will have stoichiometric selective effects as demonstrated by the *in vivo* and *in vitro* effects of drugs at the related nicotinic acetylcholine α4β2 receptors^34^,^35^.

In this study, we evaluated the effects of GABA, Zn^2+^, zolpidem and diazepam at binary α1β3 GABA_A_ receptors expressed as 3α1:2β3 and 2α1:3β3 subunit stoichiometries in *Xenopus* oocytes using two electrode voltage clamp electrophysiology. Both receptors expressed readily, and were distinguished from each other by their pharmacology. The 2α1:3β3 receptor was highly sensitive to GABA and Zn^2+^, while the 3α1:2β3 receptor was less sensitive to GABA and Zn^2+^. Interestingly zolpidem and diazepam were potent allosteric modulators of 3α1:2β3 receptors and the effects were attenuated by flumazenil. While zolpidem displayed high efficacy at the 3α1:2β3 stoichiometry, the efficacy of diazepam was significantly lower. No modulation by either zolpidem or diazepam was detected at 2α1:3β3 receptors. These results demonstrate that the α1-α1 interface contains a binding site for zolpidem mimicking the classical α1-γ2 benzodiazepine site. This novel target may explain why zolpidem enhances tonic GABA currents as binary αβ receptors are reported to be extrasynaptic, and may be a target for its motor effects after severe brain damage.

## Results

### Different α1β3 receptors are expressed by injecting *Xenopus* oocytes with different cRNA ratios

The assembly of Cys-loop receptors in *Xenopus laevis* oocytes into specific stoichiometries can be directed by injecting cRNA with variant subunit ratios^34^,^36^. To determine whether varying the injection ratios led to the expression of different receptors, we injected five mixtures of α1 and β3 cRNAs into oocytes (α1 + β3 in 1:1, 5:1, 10:1, 20:1 and 30:1 ratios). To allow for comparisons between injection ratios, all the cRNA was derived from a single stock. The total amount of cRNA injected ranged from 2.5 to 4.0 ng/oocyte and the functional properties of receptors including holding currents, GABA sensitivity and maximum peak current amplitudes were compared.

**Figure 1:**
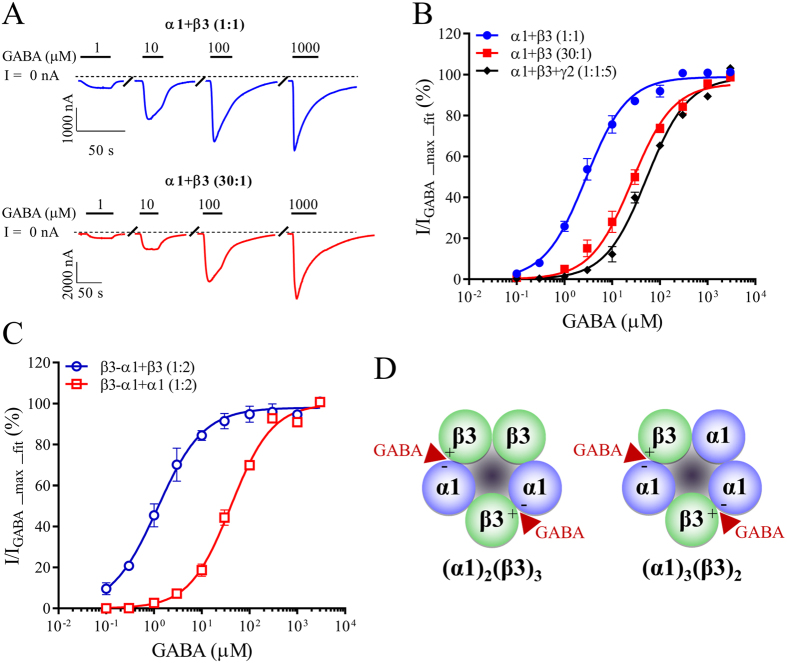
GABA-evoked responses at α1β3 and α1β3γ2 GABA_A_ receptors. *Xenopus laevis* oocytes were injected with cRNA and subjected to two-electrode voltage-clamp electrophysiology as described in the methods. For experimentation, oocytes were clamped at −60 mV and full GABA concentration response relationships were obtained on each oocyte. (**A**) Representative GABA-evoked traces from oocytes injected with the denoted cRNA mixtures. Bars above each trace indicate application periods and GABA concentrations and “/” a wash period. Dotted lines indicate a 0 nA baseline and holding currents were −150 ± 93 nA, n = 6 for α1 + β3 (1:1) and −13 ± 10 nA, n = 9 for α1 + β3 (30:1). (**B,C**) Baseline subtracted peak current amplitudes for full GABA concentration-response curves at oocytes injected with the indicated cRNA mixtures using free subunits (**B**) or a concatenated β3-α1 construct (**C**) were fitted to the Hill equation using non-linear regression (fixed bottom of 0 and slope of 1) and normalized to the maximal fitted value (I_GABA_max_fit_). Averaged normalized data points are depicted as means ± S.E. as a function of the GABA concentration, fitted to the Hill equation and regression results are presented in Table 1. Each data point represents experiments from n = 5–9 oocytes from ≥2 batches. (**D**) α1β3 GABA_A_ receptors can express in two stoichiometries of 2α1:3β3 (left) and 3α1:2β3 (right). The two binding sites for GABA at the β3(+)-α1(−) subunit interface are indicated by red arrowheads.

Initially, receptors expressed by the most extreme injection ratios 1:1 and 30:1, were compared. The holding currents of oocytes injected with α1 + β3 (1:1) cRNA were −150 ± 93 nA, n = 6 when voltage-clamped at −60 mV, suggesting constitutive receptor activity (Fig. 1A). In contrast, oocytes injected with α1 + β3 (30:1) had negligible holding current levels that averaged −13 ± 10 nA, n = 9 (Fig. 1A; p < 0.05).

GABA activated receptors expressed from both injection ratios in a concentration-dependent manner, with maximum similar peak-current amplitudes ranging from 1200 to 4200 nA (p > 0.05). Fitting peak-current amplitudes as a function of the GABA concentration to the Hill equation revealed higher GABA sensitivity with receptors from the 1:1 cRNA ratio compared with those formed from the 30:1 ratio. The derived EC_50_ values were significantly different (p < 0.0001) with a 10-fold difference between receptors at the 1:1 and 30:1 ratios (2.8 μM and 26 μM, respectively) (Fig. 1B; Table 1). The GABA sensitivity from the 30:1 injection ratio was similar to that of the ubiquitous α1β3γ2 receptor (Fig. 1B; Table 1).

In contrast, receptors formed from 5:1, 10:1, and 20:1 cRNA injection ratios resulted in less uniform receptor populations. This resulted in intermediate GABA sensitivities, concentration-response curves with shallow Hill slopes and comparatively high standard errors for individual data points (data not shown). Thus, receptors formed from different injection ratios had different functional properties that are likely the result of the expression of distinct receptor populations with altered subunit stoichiometries.

### Expressing stoichiometry specific α1β3 receptors using concatenated constructs

To determine whether data obtained by varying cRNA ratios are from uniform receptor populations that consist of 2α:3β and 3α:2β stoichiometries, the functional properties of receptors expressed with a concatenated construct linking the β3 and α1 subunits were compared to receptors expressed with the 1:1 or 30:1 cRNA injection ratios. For this experiment, the N-terminal of the α1 subunit was linked to the C-terminal of the β3 subunit to generate the β3-α1 concatenated construct as previously described^37^. When cRNA transcribed from the β3-α1 construct was injected into oocytes (2.5 ng/oocyte) no GABA-elicited currents were observed (n = 10). Therefore, to express receptors in a 2α:3β and 3α:2β stoichiometry, β3-α1 was co-injected with either the β3 or α1 subunit, respectively.

First, the properties of 2α:3β receptors were compared to receptors formed by the 1:1 injection ratio. Both holding current levels and the GABA sensitivities of oocytes expressing receptors from the concatenated β3-α1 + β3 (1:2) construct were similar to oocytes injected with free α1 + β3 (1:1) subunits, with holding currents of −153 ± 65 nA, n = 6 compared to −150 ± 93 nA, n = 6 when voltage-clamped at −60 mV. The constitutive current observed with both α1 + β3 (1:1) and β3-α1 + β3 (1:2) could be the result of homomeric β3 receptors being expressed. To determine whether or not the observed holding currents are due to homomeric β3 receptors, we evaluated oocytes expressing α1 + β3 (1:1) and β3-α1 + β3 (1:2) using histamine (1 mM), an agonist that activates only homomeric β3 receptors^38^,^39^. We found that all cells that exhibited a significant holding current injected with either α1 + β3 (1:1) (4 out of 4 cells) or β3-α1 + β3 (1:2) (1 out of 4 cells) had some response to histamine (data not shown), indicating that homomeric β3 receptors are the major contributor to the holding current. Irrespectively, GABA activated expressed receptors in a concentration-dependent manner, with maximum peak-current amplitudes ranging from 1000 to 2000 nA. Indistinguishable EC_50_ values of 1.4 μM and 2.8 μM were obtained from oocytes injected with β3-α1 + β3 (1:2) and α1 + β3 (1:1), respectively (p > 0.05, Fig. 1B,C, Table 1).

Next, properties of 3α:2β stoichiometry receptors were compared to receptors formed by the 30:1 injection ratio. Holding currents of oocytes injected with either β3-α1 + α1 (1:2) or free α1 + β3 (30:1) subunits were negligible with values of −22 ± 8 nA, n = 7 and −13 ± 10 nA, n = 9, respectively. GABA activated expressed receptors in a concentration-dependent manner, with maximum peak-current amplitudes ranging from 1400 to 2900 nA. The GABA EC_50_ values of 41 μM and 26 μM from β3-α1 + α1 (1:2) and α1 + β3 (30:1) injections were similar (p > 0.05, Fig. 1B,C, Table 1).

Thus, there were no observable differences in the functional properties of α1β3 receptors in either the 2α:3β or 3α:2β stoichiometries between receptors expressed by different subunit ratios or a concatenated construct (Fig. 1D). Although the chosen cRNA ratios for the individual subunits appeared sufficient to ensure predominantly uniform receptor populations, the use of concatenated receptors may potentially yield more uniform populations. However, this methodology inherently carries a risk that the physical linkage of subunits could affect receptor pharmacology. Hence, for the remainder of the manuscript, conclusions will be drawn from data generated using both methodologies.

### α1β3 receptors show stoichiometry-specific sensitivity to Zn^2+^ ions

Zn^2+^ ions are used to differentiate α1β3 from α1β3γ2 GABA_A_ receptors^25^. Both α1-β3 and β3-β3 interfaces contribute to the binding pockets for Zn^2+ ^40. Since 3α1:2β3 receptors do not contain a β3-β3 interface, sensitivity to Zn^2+^ will likely be altered as a result of differing subunit stoichiometries. Therefore, Zn^2+^ inhibition of α1β3 receptor currents elicited by GABA (at EC_50_ concentrations) was measured in oocytes.

**Figure 2:**
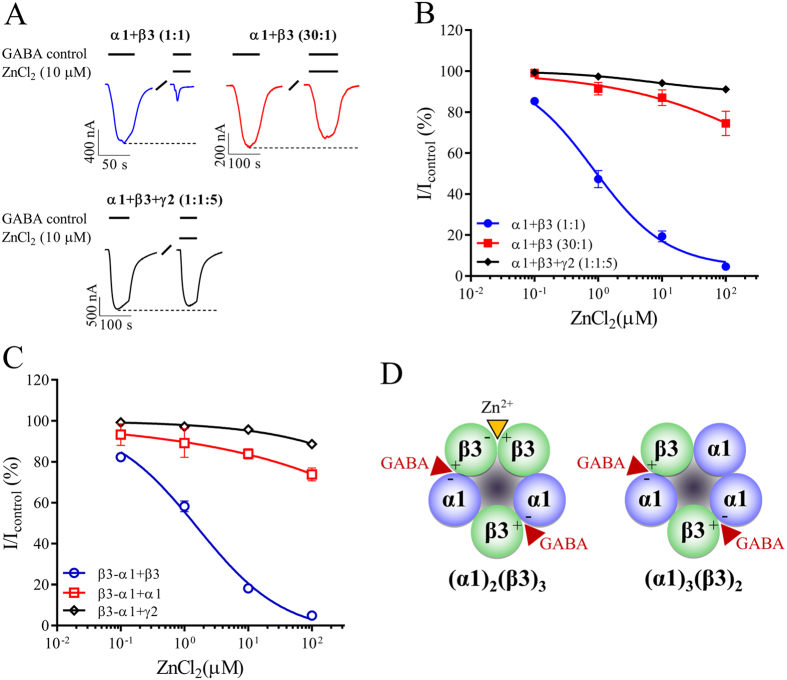
Zn^2+^ inhibition of GABA-evoked currents from α1β3 and α1β3γ2 GABA_A_ receptors. *Xenopus laevis* oocytes were injected with cRNA and subjected to two-electrode voltage clamp electrophysiology as described in the methods. Control currents (I_control_) were evoked using a GABA concentration corresponding to ~EC_50_ and inhibition by Zn^2+^ was evaluated by co-applications with GABA_control_. (**A**) Representative GABA-evoked current traces from oocytes injected with the denoted cRNA mixtures. Bars above each trace indicate GABA and Zn^2+^ application periods. Dotted lines indicate the peak current amplitude by GABA_control_ and “/” a wash period. (**B,C**) Concentration-response relationships of Zn^2+^ inhibition of GABA_control_-evoked currents at α1β3 or α1β3γ2 receptors stemming from injecting the indicated cRNA mixtures using free subunits (**B**) or a concatenated β3-α1 construct (**C**). Averaged Zn^2+^ inhibition values were depicted as means ± S.E.M as a function of the Zn^2+^ concentration and fitted to the Hill equation by non-linear regression. Regression results for α1 + β3 (1:1) were IC_50_ = 0.84 (95% CI: 0.53–1.3), nH = −0.5 ± 0.32 and for β3-α1 + β3 (1:2) were IC_50_ = 1.6 (95% CI: 1.1–2.4), nH = −0.6 ± 0.06. For the remaining cRNA mixtures, Zn^2+^ inhibition at the maximal tested concentration was too low to allow for meaningful fitting. Each data point represent experiments from n = 5–8 oocytes of ≥2 batches. (**D**) The depiction of α1β3 GABA_A_ receptor stoichiometries from Fig. 1D was modified to indicate Zn^2+^ binding in the β3(+)-β3(−) subunit interface (red/orange arrowhead).

Zn^2+^ (10 μM) inhibited 87 ± 10% (n = 5) of the GABA-induced current at 2α1:3β3 receptors formed by injection of α1 + β3 (1:1) cRNA (Fig. 2A). In contrast, only 13 ± 10% (n = 6) inhibition was observed at receptors formed by injecting α1 + β3 (30:1). Next, inhibitory concentration-response relationships were performed to ascertain the potency of Zn^2+^ at each stoichiometry. At the 2α1:3β3 stoichiometry, Zn^2+^ inhibited receptors from the 1:1 ratio with an IC_50_ value of 0.84 μM (Fig. 2B). A similar IC_50_ value of 1.6 μM for Zn^2+^ inhibition at concatenated receptors was observed with β3-α1 + β3 (1:2) injection (Fig. 2C). These two IC_50_ values were not significantly different from each other (p > 0.05). At the highest concentration of Zn^2+^, both receptors were fully inhibited.

At the 3α1:2β3 receptors, the maximal tested concentration of Zn^2+^ (100 μM) only displayed partial inhibition (26 ± 17%, n = 6) at receptors from the 30:1 ratio (Fig. 2B). Similar partial inhibition was observed using the concatenated β3-α1 + α1 (1:2) construct (Fig. 2C). In both cases, the level of inhibition was too low to allow for meaningful fitting to the Hill equation. The partial inhibition of Zn^2+^ at 3α1:2β3 receptors mimicked observations at α1β3γ2 receptors (Fig. 2B,C).

These data demonstrate that receptors with a β3-β3 interface have high sensitivity to inhibition by Zn^2+^ (Fig. 2D). While it was previously suggested that residues located in the α1-β3 interface also contribute to Zn^2+^ sensitivity^40^ this appears to require substantially higher concentrations. In addition, α1β3γ2 receptors that also lack the β3-β3 interface, were likewise relatively insensitive to Zn^2+^ inhibition at concentrations below 100 μM.

### Zolpidem is a positive modulator of α1β3 with a 3α1:2β3 subunit stoichiometry

The α1-α1 interface of 3α1:2β3 receptors is homologous to the α1-γ2 interface that binds benzodiazepines and non-benzodiazepines. Zolpidem has *in vivo* effects that are not related to binding in the classical α1-γ2 benzodiazepine site, and we wanted to evaluate whether this α1 preferring modulator displayed any efficacy at α1β3 possessing an α1-α1 interface. Zolpidem (1 μM) was co-applied with a low GABA concentration (~EC_5–10_) to evaluate potential modulation of control currents at 3α1:2β3 and compared with 2α1:3β3 and α1β3γ2 receptors.

**Figure 3:**
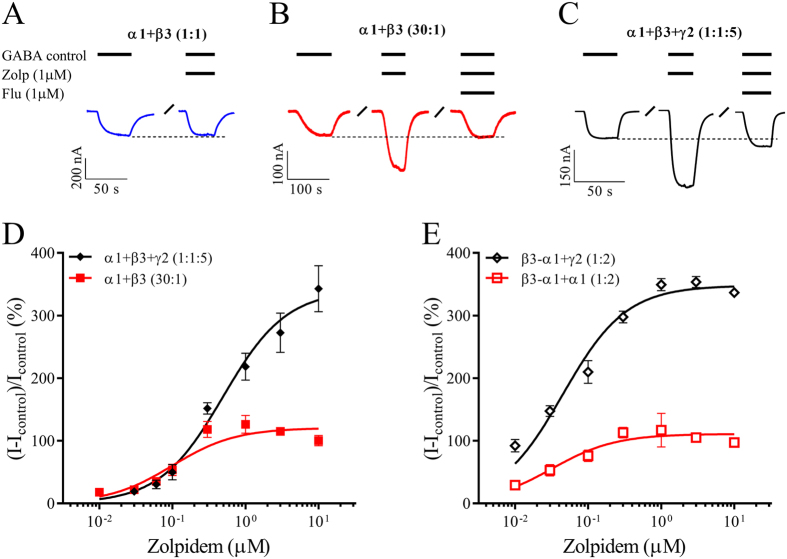
Zolpidem modulation of GABA-evoked currents from α1β3 and α1β3γ2 GABA_A_ receptors. *Xenopus laevis* oocytes were injected with cRNA and subjected to two-electrode voltage clamp electrophysiology as described in the methods. Control currents (I_control_) were evoked using a GABA concentration corresponding to ~EC_5–10_ and modulation by zolpidem was evaluated by co-applications with GABA_control_. (**A–C**) Representative GABA-evoked current traces from oocytes injected with the denoted cRNA mixtures. Bars above each trace indicate GABA, zolpidem (Zolp) and flumazenil (Flu) concentrations and application periods. Dotted lines indicate the peak current amplitude by GABA_control_ and “/” a wash period. For the specific traces, zolpidem had no robust effects at receptors from α1 + β3 (1:1) injection (**A**), but showed 130% modulation at receptors from α1 + β3 (30:1) injection which was inhibited 95% by co-application of flumazenil (**B**). Zolpidem likewise modulated receptors from injection of α1 + β3 + γ2 (1:1:5) by 160% which could be inhibited 85% by flumazenil (**C**). (**D,E**) Concentration-response relationships of zolpidem modulation of GABAcontrol-evoked currents at α1β3 or α1β3γ2 receptors stemming from injecting the indicated cRNA mixtures using free subunits (**D**) or a concatenated β3-α1 construct (**E**). Average modulatory values were depicted as means ± S.E.M as a function of the zolpidem concentration and fitted to the Hill equation by non-linear regression. Each data point represents experiments from n = 5–7 oocytes from ≥2 batches and regression results are presented in Table 1.

As expected, zolpidem (1 μM) had no effect at 2α:3β receptors expressed by a 1:1 ratio of free subunits (Fig. 3A). In contrast, 3α1:2β3 receptors expressed from the 30:1 ratio were positively modulated by zolpidem. This resulted in an increase in current amplitudes of more than 100% compared with the current elicited by the GABA alone. This enhancement was inhibited by co-application of flumazenil (1 μM), a benzodiazepine site neutral antagonist (Fig. 3B). As expected, zolpidem (1 μM) also enhanced GABA-elicited currents at α1β3γ2 receptors, an enhancement that could be inhibited by flumazenil (Fig. 3C).

To determine the potency of zolpidem potentiation at 3α1:2β3 receptors, full concentration-response relationships were obtained and compared with those from the α1β3γ2 receptor. When injecting free subunits using α1 + β3 (30:1) and α1 + β3 + γ2 (1:1:5) cRNA, zolpidem had a significantly lower EC_50_ value of 0.10 μM at α1β3 receptors compared to an EC_50_ value of 0.48 μM at α1β3γ2 (p < 0.01) (Fig. 3D; Table 2). However, when injecting cRNA containing the concatenated construct using β3-α1 + α1 (1:2) and β3-α1 + γ2 (1:2), the EC_50_ value of 0.030 μM at α1β3 receptors was not significantly different to the EC_50_ value of 0.050 μM at α1β3γ2 receptors (p > 0.05) (Fig. 3E; Table 2). Efficacy levels at α1β3 receptors were 120% and 110% for the 30:1 ratio and concatenated receptors, respectively. Higher values were observed at α1β3γ2 receptors with 340% and 350% at α1 + β3 + γ2 (1:1:5) *vs*. β3-α1 + γ2 (1:2), respectively.

Hence zolpidem modulated GABA-evoked currents at 3α1:2β3 receptors. Importantly, the potency of zolpidem at α1β3 (3α1:2β3) receptors was comparable with α1β3γ2 receptors regardless of whether free subunits or concatenated subunits were injected. While the potency of zolpidem was similar at both receptors, these experiments suggest that zolpidem enhanced α1β3γ2 receptors with greater efficacy. However, in this type of assay efficacy of a modulator is highly dependent on the utilized GABA concentration and should be treated with caution.

### Modulatory mechanism of action of zolpidem at α1β3 receptors possessing a 3α1:2β3 subunit stoichiometry

The hallmark feature of allosteric modulation via the α1-γ2 benzodiazepine site by *e.g*. diazepam and zolpidem is an increase in the apparent potency of GABA. This causes a shift in the GABA concentration-response curve to the left in presence of zolpidem with little accompanying change in the maximal current amplitudes. In order to assess how zolpidem affects 3α1:2β3 stoichiometry, GABA concentration-response relationships were measured in the presence of zolpidem and compared to that in its absence.

**Figure 4:**
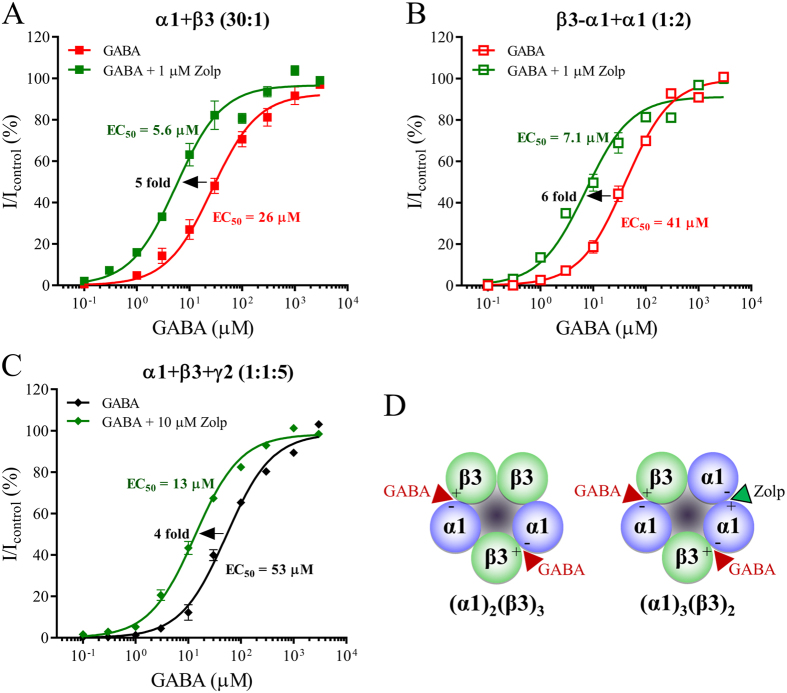
Mechanism of zolpidem modulatory actions at α1β3 and α1β3γ2 GABA_A_ receptors. *Xenopus laevis* oocytes were injected with cRNA and subjected to two-electrode voltage clamp electrophysiology as described in the methods. (**A–C**) Full GABA concentration-response relationships were obtained in presence of the indicated concentrations of zolpidem (Zolp) at receptors stemming from injection of cRNA of free subunits (**A,C**) or a concatenated construct (**B**). Baseline subtracted GABA + zolpidem peak current amplitudes were normalized to a maximal GABA control response (3 mM) in the same oocytes. Averaged normalized data points are depicted as means ± S.E.M. as a function of the GABA concentration and fitted to the Hill equation with regression results are presented in Table 1. Each data point represents experiments from n = 5–7 oocytes from ≥2 batches. GABA concentration response relationships in absence of zolpidem (from Fig. 1) are included for comparison. (**D**) The depiction of α1β3 GABA_A_ receptor stoichiometries from Fig. 2D was modified to indicate zolpidem binding in the α1(+)-α1(−) subunit interface (red/green arrowhead).

At 3α1:2β3 receptors expressed by injecting α1 + β3 (30:1) cRNA, zolpidem (1 μM) left-shifted the GABA concentration-response curve by decreasing the EC_50_ value from 26 μM to 5.6 μM (Fig. 4A, Table 1). Hence, zolpidem modulation caused a significant 5-fold change (p < 0.0001) of the GABA potency with no observed change in the maximum GABA-evoked peak current amplitudes. At receptors obtained by the concatenated β3-α1 + α1 (1:2) construct, zolpidem (1 μM) likewise caused a significant (p < 0.0001) 6-fold change in the potency of GABA, with an increase in the EC_50_ value from 41 μM to 7.1 μM (Fig. 4B, Table 1). At α1β3γ2 receptors, zolpidem (10 μM) significantly decreased the EC_50_ value from 53 μM to 13 μM (Fig. 4C; Table 1; p < 0.0001) with no change in the maximum peak current amplitudes.

These data demonstrate that the mechanism of modulatory action by zolpidem is increase of the GABA potency at both α1β3 and α1β3γ2 receptors. This increase ranged from 4 to 6 fold, which is in agreement with previous observations at α1β3γ2 receptors^8^. The modulatory effect was not accompanied by any change in maximal GABA-evoked peak current amplitudes and the similar magnitude of the changes in EC_50_ values suggest that zolpidem has a similar efficacy at the two receptor types. Although zolpidem is not structurally a classical benzodiazepine, it binds at a similar site within the α1-γ2 of α1βγ2 receptors as benzodiazepines. Taken together, zolpidem is most likely binding at the α1-α1 interface of 3α:2β receptors to modulate receptor function analogous to the modulation of receptor function via binding at the α1-γ2 interface (Fig. 4D).

### Diazepam enhances GABA currents at α1β3 receptors with a 3α1:2β3 subunit stoichiometry albeit with low efficacy

**Figure 5:**
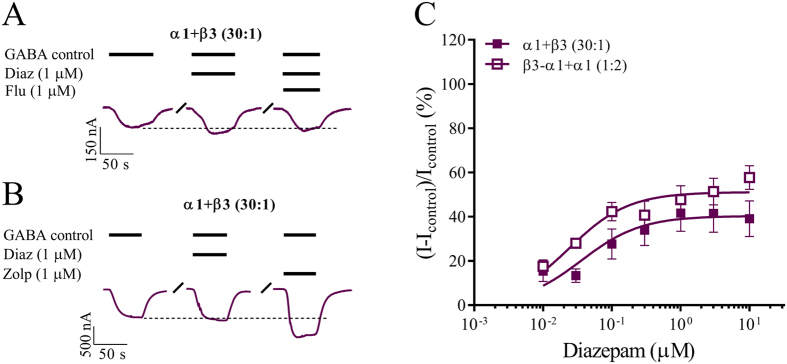
Diazepam modulation of GABA-evoked currents from α1β3 GABA_A_ receptors. *Xenopus laevis* oocytes were injected with cRNA and subjected to two-electrode voltage clamp electrophysiology as described in the methods. Control currents (I_control_) were evoked using a GABA concentration corresponding to ~EC_5–10_ and modulation by diazepam was evaluated by co-applications with GABA_control_. (**A,B**) Representative GABA-evoked current traces from oocytes injected with the denoted cRNA mixtures. Bars above each trace indicate GABA, diazepam (Diaz) and flumazenil (Flu) concentrations and application periods. Dotted lines indicate the peak current amplitude by GABA_control_ and “/” a wash period. (**C**) Concentration-response relationships of diazepam modulation of GABAcontrol-evoked currents at α1β3 receptors stemming from injecting the indicated cRNA mixtures using free subunits or a concatenated β3-α1 construct. Averaged modulatory values were depicted as means ± S.E as a function of the diazepam concentration and fitted to the Hill equation by non-linear regression. Each data point represents experiments from n = 6 oocytes from ≥2 batches. Regression results with 95% confidence intervals for α1 + β3 (30:1) were: EC_50_ value of 0.040 μM (0.010–0.12) and E_max_ value of 40% (33–48) whereas results for β3-α1 + α1 (1:2) were: EC_50_ value of 0.020 μM (0.010–0.04) and E_max_ value of 51% (46–56).

Finally, we determined whether α1β3 (3α1:2β3) receptors could be modulated by the classical benzodiazepine diazepam, which binds with equal affinity to GABA_A_ receptors containing α1, α2, α3 and α5 subunits^41^,^42^. Like zolpidem, diazepam (1 μM) enhanced GABA-induced currents at α1β3 receptors obtained with α1 + β3 (30:1) cRNA and this effect was inhibited by co-application of flumazenil (Fig. 5A). However, the enhancement by diazepam was low in comparison with zolpidem (Fig. 5B). A full concentration-response relationship revealed an EC_50_ value for diazepam of 0.040 μM (Fig. 5C). A similar EC_50_ value of 0.020 μM was estimated for diazepam modulation of concatenated receptors and the observed potencies are similar to that reported for α1β3γ2 receptors^8^. From the concentration-response relationships, it is evident that the enhancement by diazepam displayed lower efficacy compared with zolpidem. When using free subunit cRNA diazepam enhanced GABA-elicited currents by a maximum of 40% whereas use of concatenated subunits resulted in 51% modulation and these were not significantly different from each other (Fig. 5C; p > 0.05). This represents half or less of the modulation observed with zolpidem (Table 2). Analogous to zolpidem, diazepam (1 μM) did not change the maximal GABA-evoked current amplitudes at 2α1:3β3 receptors obtained by injecting α1 + β3 (1:1) (data not shown).

## Discussion

Binary GABA_A_ receptors co-exist with their ternary counterparts^23^,^24^,^25^ and are gaining attention for their sensitivity to certain benzodiazepines^25^,^26^,^27^. When evaluating the pharmacology of binary receptors the concept of stoichiometry-specific binding sites becomes important, because each receptor stoichiometry will have a specific binding interface that will contribute to a distinct pharmacological profile for that receptor^34^,^35^,^36^. For pentameric α1β3 receptors, the third subunit position, normally occupied by a γ2 subunit in α1β3γ2 receptors, is substituted with either an α1 or a β3 subunit leading to two distinct receptors, that differ by the presence of either an α1-α1 or a β3-β3 interface (Fig. 1D). Pharmacologically targeting either the α1-α1 or β3-β3 interface leads to stoichiometric selective effects as demonstrated by the *in vivo* and *in vitro* effects of drugs at the related nicotinic acetylcholine α4β2 receptors^34^,^35^. In this study, we demonstrate that binary α1β3 receptors composed of 2α1:3β3 or 3α1:2β3 subunit stoichiometry were differentiated from each other using GABA, Zn^2+^, zolpidem and diazepam.

Two methodologies were used to express the two receptor stoichiometries (2α1:3β3 or 3α1:2β3) in *Xenopus laevis* oocytes. In the first method, we injected cRNA mixtures with variant subunits ratios, causing relative overexpression of one subunit versus the other, and hence, increasing the likelihood of receptor assembly with more of the overexpressed subunit. While this is an efficient method, it cannot always ensure uniform receptor populations or rule out the formation of “obscure” receptor populations such as 4α:1β receptors. In the second method, concatenated β3-α1 subunits were used, ensuring that linkers force the assembly process. While this is efficient for creating uniform receptor populations, the presence of linkers may affect receptor pharmacology. Nevertheless, we found that both methods enabled the assembly of α1β3 receptors, which were indistinguishable in their pharmacology using either methodology and concluded the receptors to be either 2α1:3β3 or 3α1:2β3 receptors. This contrasted the original study using concatenated β2-α1 subunits and individual α1 or β2 subunit mRNA. It is not clear why Baumann and colleagues did not see functional receptors when using β2-α1 and individual α1 subunits^43^. However the study used a 1:1 injection ratio while we used a 2-fold excess of α1 subunit mRNA. Further we used the β3 subunit and not β2 and there may be differences in the formation of receptors depending on the type of β subunit.

*Xenopus* oocytes expressing α1β3 (2α1:3β3) receptors consistently displayed voltage-clamp holding-current levels constituting approximately 10% of maximal GABA-evoked peak current amplitudes. This suggests the existence of homomeric β3 receptors contributing to the holding current as histamine was able to activate cells that exhibited constitutive currents^38^,^39^. In contrast, expression of 3α1:2β3 receptors did not result in levels significantly different from un-injected control oocytes. However, the potency of GABA differed approximately 10-fold between 2α1:3β3 and 3α1:2β3 receptors with 2α1:3β3 displaying the highest GABA sensitivity. The lower GABA sensitivity at 3α1:2β3 receptors was not significantly different to the “classical” α1β3γ2 receptor indicating “normal” receptor function.

Previous studies demonstrated that Zn^2+^ inhibits binary GABA_A_ α1β3 receptor function by an allosteric mechanism and this action is critically dependent on the composition of α1 and β3 subunits^40^,^44^. This is consistent with our data showing that binary GABA_A_ receptors can be differentiated by Zn^2+^, showing that 2α1:3β3 receptors are easily blocked by Zn^2+^ while 3α1:2β3 receptors are not. Thus, the presence of an extra β3 subunit, and thereby a β3-β3 interface, confers the resulting binary receptors with several unique characteristics such as, higher sensitivity for GABA and higher sensitivity for Zn^2+^ inhibition. The high sensitivity to Zn^2+^ inhibition is intriguing given their extrasynaptic location. Physiological brain concentrations of Zn^2+^ can reach up to 100 μM^45^,^46^,^47^,^48^, *i.e*. the sweet-spot for completely inhibiting 2α1:3β3 receptors, and one could speculate that these receptors may in part be responsible for a Zn^2+^-regulated tonic current.

In contrast, binary receptors with an α1-α1 interface have more resemblance to ternary α1β3γ2 receptors. They exhibit a lower degree of constitutive activity, and lower sensitivity for GABA and Zn^2+^ inhibition. Furthermore, we demonstrate that zolpidem efficiently modulates GABA activity at 3α1:2β3 but not 2α1:3β3 receptors with a binding site for zolpidem most likely at the α1-α1 interface. Several lines of evidence point to zolpidem’s actions occurring via the α1-α1 interface of the 3α1:2β3 receptor in a manner that mimics the potentiation of zolpidem via binding to the classical α1-γ2 benzodiazepine site: i) Like zolpidem, diazepam is a positive allosteric modulator of 3α1:2β3 receptors albeit with lower efficacy levels; ii) the effects of zolpidem and diazepam are inhibited by the antagonist flumazenil at a concentration normally used to inhibit benzodiazepines at the α1-γ2 benzodiazepine site; and iii) the modulatory effect of zolpidem at 3α1:2β3 receptors was one of increasing the potency of GABA without significant change to maximal current amplitudes. These data comprise signature features of modulatory actions by benzodiazepines via the classical α1-γ2 interface benzodiazepine site^4^,^10^,^11^, however, as such a site is not available in 2α1:3β3 receptors, zolpidem’s actions must occur via the α1-α1 interface. Interestingly, the potency of zolpidem at 3α1:2β3 receptors was equal to or marginally higher than that observed at α1β3γ2 receptors suggesting that the observed effects could have clinical relevance.

In addition to phasic currents, zolpidem modulates tonic GABA currents in the rat dorsal motor nucleus of the vagus^20^ and primary motor cortex^15^. This is intriguing as the subunit composition of zolpidem-sensitive extrasynaptic receptors is unlikely to be due to α5, γ1/3 or δ containing GABA_A_ receptors, as such receptors do not significantly respond to zolpidem^8^,^21^. Recently, α1β3γ2 receptors were reported to exist at extrasynaptic sites^49^ but the residual effects of zolpidem in γ2F77I knockin mice excludes these receptors as a target for zolpidem. Although non-GABA_A_ receptors may also contribute to zolpidem’s effects, binary α1β3 receptors represent 10% of the total number of extrasynaptic receptors in hippocampal pyramidal neurons^25^. Thus binary α1β3 receptors that possess an α1-α1 subunit interface may contribute to the residual zolpidem effects reported in γ2F77I knockin mice that lack zolpidem–sensitive α-γ2 interfaces^19^,^50^,^51^.

Binary α1β3 receptors that are positively modulated by zolpidem may also have significant clinical effects in a number of neurological conditions where the ratio and location of various GABA receptor subunits are altered, for example, stroke^52^,^53^,^54^, or epilepsy^55^,^56^. Under these conditions, dramatic changes are occurring in the brain. After stroke the α1 mRNA significantly increases^52^,^53^,^54^, resulting in an increase in α1 protein^52^. At the same time β-subunit mRNA decreases, and thus may affect the formation of GABA_A_ receptors in a way that the subunit composition of the receptors is altered favouring receptors that possess an α1-α1 subunit interface. In order to ascertain that the increase in α1 protein after stroke results in a change in receptor function that mimics the pharmacology of binary α1β3 receptors possessing an α1-α1 interface, further work including patch clamp recordings are required.

In corroboration, there are increasing clinical reports documenting that zolpidem is not a typical hypnotic, improving cognitive and motor function in human patients with severe brain injury. For instance, acute administration of zolpidem in a patient suffering from brain injury resulted in a transient improvement in aphasia^57^. Brefel-Courbon and colleagues (2007) reported improved motor performance and neuropsychological status in a patient who was given zolpidem^58^. In addition, zolpidem has benefits on motor disorders in patients with Parkinson’s disease^13^,^14^,^15^, has a transient improvement of spinocerebellar ataxia^16^, and improve symptoms of progressive supranuclear palsy^17^. Whilst flumazenil inhibits the effect of zolpidem indicating a GABA_A_ receptor is involved with these patients^59^, classical benzodiazepines are not very effective in improving speech or motor function^17^ indicating an unusual GABA_A_ receptor to be the target.

Similar positive modulation is seen by zolpidem at α1-α1 and α1-γ2 interfaces, however the complete molecular interactions involving ligand binding and the accompanying conformational changes to the receptor complex still needs to be resolved. While the principal face (+) of the α1 subunit is the same in the two sites, the complementary face (−) of α1 and γ2 are obviously different. Although the actions of zolpidem, diazepam and flumazenil suggest substantial overlap between the two sites, other compound structures could potentially be fully selective for the α1-α1 interface. Future studies are required to ascertain the structure-function requirements for this interface.

In conclusion, this study demonstrates GABA_A_ α1β3 receptors can express in either 2α1:3β3 and 3α1:2β3 stoichiometry possessing either a β3-β3 or α1-α1 interface, respectively. This difference leads to stoichiometry-specific binding sites, fundamentally changing receptor pharmacology. Indeed, zolpidem at clinically relevant concentrations enhanced GABA actions at 3α1:2β3 but not 2α1:3β3 receptors by binding to the α1-α1 interface in a manner mimicking its actions at the classical benzodiazepine site. These receptors may be the molecular target for zolpidem’s described extrasynaptic and clinical effects and constitute a potentially interesting drug target for neurological conditions (*e.g*. stroke and epilepsy) where the α1 mRNA and protein levels are significantly upregulated.

## Methods

### Chemicals

GABA (4-aminobutanoic acid), flumazenil (Ethyl 8-fluoro-5-methyl-6-oxo-5,6-dihydro-4*H*-benzo[*f*]imidazo[1,5-*a*][1,4]diazepine-3-carboxylate), pyruvate, theophylline, gentamycin and zinc chloride were obtained from Sigma Aldrich, Sydney, Australia. Diazepam was obtained from Apin Chemicals Ltd, Oxon, UK, and zolpidem (*N*,*N*-dimethyl-2-(6-methyl-2-*p*-tolylimidazo[1,2-a]pyridin-3-yl) acetamide) from Chemieliva Pharmaceutical, Chongqing, China. Tricaine was purchased from Western Chemical, USA.

### GABA_A_ receptor subunit cRNA

Human α1 and γ2 cDNA subcloned in pcDM8 and β3 in pGEMHE, were linearized with the appropriate restriction endonucleases (NotI for α1 and γ2, NheI for β3 subunit, respectively). Concatenated β3-α1 subunits were developed as previously described^43^, subcloned in pNS3z and linearlized with NotI. cRNA was produced from linearized plasmids using the ‘mMessage mMachine’ T7 transcript kit from Ambion (Austin, TX, USA) as previously described^60^,^61^. A total of 2–4 ng of cRNA was injected per oocyte. When using free subunits of α1, β3 and γ2, cRNAs were mixed in variant ratios. To ensure incorporation of a free subunit in the pentameric complex, concatenated β3-α1 subunit cRNA was injected with either α1 or γ2 cRNAs in a ratio of 1:2. However to avoid any potential formation of β3 homomeric receptors concatenated β3-α1 subunit cRNA was injected with β3 in a 1:1 ratio.

### *Xenopus* oocyte extraction and preparation

All procedures were using *Xenopus laevis* frogs were approved by the animal ethics committee of The University of Sydney (AEC No. 2013/5269) and are in accordance to the National Health and Medical Research Council (NHMRC) of Australia. In brief, *Xenopus laevis* were anaesthetized using 0.2% w/v solution of tricaine methanesulfonate or tricaine-S (Western Chemical, USA). A transverse incision of about 3 mm in length was made through the outer layer of her skin on the lateral ventral surface. Another incision was made through the connective tissue and muscle layer to reach the ovary wall. A section of the ovary was carefully removed onto the surface of the frog. A small section of ovary was separated and transferred to a tube containing the OR2 solution (82.5 mM NaCl, 5 mM HEPES, 2 mM MgCl_2_ and 2 mM KCl; pH 7.4). Oocytes were separated manually from their follicles and digested using 40 mg collagenase A diluted in 15 ml OR2 solution at 18 °C for about 1 hour until the oocytes were fully detached from the follicles and the ovary tissue.

Stage V-VI oocytes were injected with 50.6 nl cRNA solution composed of GABA_A_Rs subunit cRNAs in the required ratio. The injected oocytes were incubated in ND96 solution (96 mM NaCl, 5 mM HEPES, 2 mM MgCl_2_, 1 mM KCl and 1.8 mM CaCl_2_; pH 7.4) supplemented with 2.5 mM Na-pyruvate, 0.5 mM theophylline, 50 mg/ml gentamycin and 50 mg/ml tetracycline for 2–5 days at 18 °C.

### Two-electrode voltage-clamp electrophysiology

The electrophysiological experiments were performed by the two-electrode voltage clamp technique. The membrane potential was measured using an Oocyte Clamp OC-725C amplifier (Warner Instruments Corp, CT, USA) by the voltage electrode with current simultaneously injected by the current electrode to maintain the potential difference across the oocyte membrane at −60 mV. Data were acquired with a LabChart v. 3.5.2 analogue to digital converter and currents low-pass-filtered at 1 kHz and sampled at 3 kHz were measured offline with LabChart v. 3.5.2 software. The bath solution contained the ND96 solution and electrodes were filled with solution of 3 M KCl (0.5–2 MΩ). Solutions were bath applied using a gravity-fed perfusion system running at 5 ml/min.

### Data Analysis

For Zn^2+^ inhibition studies, control currents (I_control_) were evoked using a GABA concentration corresponding to ~EC_50_ and inhibition by Zn^2+^ was evaluated by co-applications with GABA_control_. Averaged Zn^2+^ inhibition in the presence of GABA EC_50_ were depicted as means ± S.E.M. as a function of the Zn^2+^ concentration and fitted to the Hill equation by non-linear regression. Enhancement of GABA-gated Cl^−^ currents was measured by co-applying modulators with a GABA concentration that elicited 5% of the maximal current amplitude as determined at the beginning of each experiment. The enhancement of the GABA-gated Cl^−^ current (I_GABA_) was defined as I = I_max_/(1 + [EC_50_/(A)]^*n*^H); A is the agonist concentration, I is the current and I_max_ is the maximum current, EC_50_ is the concentration of GABA that produces a response that is 50% of the maximum current, *n*_H_ is the Hill Coefficient. EC_50_ values are expressed as mean with 95% confidence intervals and Hill coefficients (*n*_H_) are expressed as mean ± S.E.M.

Concentration–response curves were generated and the data fitted by non-linear regression analysis using GraphPad Prism software (version 5.0). Statistical significance was calculated using unpaired Student *t*-test with a confidence interval of p < 0.05 to compare parameters derived from individual experiments and data given as mean with 95% confidence intervals from at least 5 oocytes and at least 2 different batches. The logEC_50_, derived from individual comparisons, were used for statistical comparisons.

## Additional Information

**How to cite this article**: Che Has, A. T. *et al*. Zolpidem is a potent stoichiometry-selective modulator of α1β3 GABA_A_ receptors: evidence of a novel benzodiazepine site in the α1-α1 interface. *Sci. Rep*. **6**, 28674; doi: 10.1038/srep28674 (2016).

## Figures and Tables

**Table 1:** GABA concentration response relationships at various GABA_A_ receptors.

Receptor	cRNA ratio	EC_50_ (μM)	E_max_ (%)	n
α1 + β3	1:1	2.8 (2.4–3.3)	99 (96–102)	6
α1 + β3	30:1	26 (21–32)	96 (91–100)	9
α1 + β3 + γ2	1:1:5	53 (45–61)	99 (96–102)	5
α1 + β3 (1 μM Zolpidem)	30:1	5.6 (4.5–7.0)	97 (93–100)	5
α1 + β3 + γ2 (10 μM Zolpidem)	1:1:5	13 (12–15)	98 (96–100)	5
β3-α1 + β3	1:2	1.4 (1.2–1.8)	98 (94–101)	6
β3-α1 + α1	1:2	41 (35–48)	100 (97–103)	7
β3-α1 + α1 (1 μM Zolpidem)	1:2	7.1 (5.7–9.0)	91 (87–95)	7

*Xenopus laevis* oocytes were injected with cRNA mixtures containing the indicated GABA_A_ receptor subunits. Background subtracted peak current amplitudes for full GABA concentration-response curves in presence or absence of zolpidem were fitted to the Hill equation (fixed bottom of 0 and slope of 1) using non-linear regression and normalized to the maximal fitted value. Averaged normalized data points were next fitted to the Hill equation and resultant EC_50_ values and maximal efficacies (E_max_) are presented as mean with 95% confidence intervals for n experiments.

**Table 2:** Zolpidem modulation of GABA-evoked currents at various GABA_A_ receptors.

Receptor	cRNA ratio	EC_50_ (μM)	E_max_ (%)	*n*
α1 + β3	30:1	0.10 (0.06–0.16)	120 (110–130)	5
α1 + β3 + γ2	1:1:5	0.48 (0.31–0.78)	340 (300–380)	6
β3-α1 + α1	1:2	0.032 (0.015–0.070)	111 (96–126)	9
β3-α1 + γ2	1:2	0.045 (0.035–0.057)	348 (333–363)	5

*Xenopus laevis* oocytes were injected with cRNA mixtures containing the indicated GABA_A_ receptor subunits. Zolpidem was co-applied with a control concentration of GABA corresponding to an EC_5–10_ value. Modulatory efficacies of zolpidem were calculated as percentage change from the GABA_control_-evoked currents. Averaged efficacies were next fitted to the Hill equation (fixed bottom of 0 and slope of 1) and resultant EC_50_ values and maximal efficacies (E_max_) are presented as mean with 95% confidence intervals for *n* experiments.
